# Challenges and Weaknesses of Leadership and Governance-related Health Policies in Iran: A Systematic Review

**DOI:** 10.34172/aim.28907

**Published:** 2024-09-01

**Authors:** Rahim Khodayari-Zarnaq, Khorshid Mobasseri, Shabnam Ghasemyani, Fatemeh Sadeghi-Ghyassi, Maryam Naghshi, Neda Kabiri

**Affiliations:** ^1^Department of Health Policy and Management, School of Management and Medical Informatics, Tabriz University of Medical Sciences, Tabriz, Iran; ^2^Department of Geriatric Health, Faculty of Health Sciences, Tabriz University of Medical Sciences, Tabriz, Iran; ^3^Department of Health Service Management, School of Health Management and Information Sciences, Iran University of Medical Sciences, Tehran, Iran; ^4^Research Center for Evidence-based Medicine, Iranian EBM Centre: A JBI Centre of Excellence, Faculty of Medicine, Tabriz University of Medical Sciences, Tabriz, Iran; ^5^Medical Philosophy and History Research Center, Tabriz University of Medical Sciences, Tabriz, Iran

**Keywords:** Governance, Health policy, Iran, Leadership, Systematic review

## Abstract

**Background:**

A better understanding of health system performance requires evaluating achievements and challenges, thereby providing a basis for effective reforms. This systematic review aims to investigate the challenges and weaknesses of leadership and governance-related health policies in Iran.

**Methods:**

In this qualitative systematic review, we followed the instructions of the Joanna Briggs Institute (JBI). It encompassed qualitative studies assessing challenges and weaknesses of leadership and governance-related health policies. Thematic synthesis was conducted in three stages to identify common themes.

**Results:**

The primary database search yielded 1890 records, of which 152 were fully assessed, resulting in the inclusion of 57 studies in this review. Thematic synthesis produced 157 structured codes and identified 11 main descriptive themes of challenges in leadership and governance-related health policies. These themes included hospital autonomy policy, challenges to the entire health system, governance of medical universities, healthcare payment systems, sustainable universal health insurance coverage, informal payments, insurance systems, induced demand, strategic purchasing of health services, the family physician program, family physician and rural health insurance programs, and primary healthcare human resources.

**Conclusion:**

The identified challenges underscore the urgent need for strategic reforms and interventions to overcome the complex issues plaguing the healthcare system. By addressing these challenges, policymakers and top healthcare managers might ensure that the population have access to high-quality care in a more responsive healthcare system.

## Introduction

 Health is universally regarded as a fundamental human right, and as such, all individuals should have access to the necessary resources for healthcare.^1^ Over the past decade, the enhancement of health systems has emerged as a top priority for nations.^2^ The increasingly intricate, multifaceted, and demanding societal needs, particularly in the realm of health, necessitate a responsive public system. It is imperative to continuously monitor, review, and reform health systems globally in order to bolster the efficiency and effectiveness of healthcare provision, ensure quality and equality, and establish sustainable financing.^3^

 The World Health Organization defines reforms as substantial endeavors with specific objectives geared towards enhancing the health system’s performance, prompting countries to regularly review policies and procedures.^4^ Healthcare systems in various countries have undergone numerous reforms to address challenges and enhance health-related processes, demonstrating notable progress in areas such as health promotion. Nevertheless, none of these reforms have uniformly achieved their intended goals.^5-9^

 In numerous countries, these reforms have yielded positive results for their respective health systems.^10^ Over recent decades, Iran’s health system has undergone significant transformations in response to social, economic, and technological advancements, aiming to achieve comprehensive health coverage. Reforms in the structure and organization of the health system include primary healthcare system establishment in 1980, medical education integration with healthcare services in 1985, introduction of the universal health insurance law in 1994, establishment of the novel system for managing hospitals in 1995, implementation of family physician program in 2005, and finally, implementation of the health transformation plan in 2013. These initiatives have encountered both challenges and successes.^11-21^

 In recent years, the Iranian health system has implemented various measures aimed at providing accessible healthcare for Iranian citizens and bolstering the overall health system.^1,22-24^ However, the health reforms undertaken in Iran over the past three decades have revealed that several initiatives have faced challenges, largely attributed to political developments in the country.^20,22^ While the expansion of primary healthcare in the 1980s, grounded in the principles of health for all, and the establishment of the health and treatment network in 1984 have been notable successes of the Iranian healthcare system, the accessibility of services at the secondary and tertiary levels has not shown improvement. In reality, the benefits have been confined to the realm of primary healthcare, and the country’s medical care system continues to grapple with a weak referral system.^1,22,25^

 Following the enactment of the “Universal Health Insurance Law” in 1994, measures were identified and implemented to expand health coverage among population and to provide financial assistance for healthcare expenses.^25^ The aim of the family physician program was improving the health network and enhancing the referral system through the mechanism of family physician as a gatekeeper, although it still falls short of the ideal.^20,26^ In the most recent effort to reform the health system, the 11th government initiated a series of reforms in 2013 to facilitate universal health coverage, extend financial support to households, improve equity in access to healthcare services for community members, and enhance the overall efficiency of the health system.^27^

 The Health Transformation Plan was implemented through several phases, aligning with the fifth national health development strategies for a 5-year period (2011‒2016) and new strategies aimed at achieving universal coverage by 2025 and providing comprehensive health services.^26,28-32^ The plan encompasses goals such as ensuring sustainability in financial resources in the health sector, reducing direct costs for hospitalized patients, offering financial protection against health expenses, enhancing access to health services and high-quality facilities, improving the provision of health services, and fostering further advancements in the health sector. Various interventions have been undertaken to realize these objectives.^24,31^

 However, the plan has faced challenges such as inadequate sustainable financing, the neglect of primary and preventive healthcare, and the marginalization of patients in private hospitals, which have had a detrimental impact.^33^ Furthermore, the bankruptcy of insurance companies, reduction in the quality of services, lack of sustainable financing, neglect of primary and preventive healthcare, and the marginalization of patients in private hospitals are among the challenges faced by this plan after six years of implementation.^25,34^ Despite these challenges, the project has achieved significant milestones, including enhanced capacity to deliver health services, reduction of informal payments and out-of-pocket expenses, decreased patient visits for purchasing medicine and consumables/equipment, expansion of insurance coverage, and increased employee satisfaction.^34^

 To enhance the understanding of health system performance, it is crucial to assess facilitators, barriers, and challenges of health systems initiatives.^25^ Given the lack of comprehensive investigation into the obstacles and challenges of implemented health policies, in this study, we systematically evaluated the impediments, challenges, and weaknesses of leadership and governance-related health policies in Iran.

## Materials and Methods

 This study was conducted to summarize the challenges and weaknesses in leadership and governance-related health policies in the Iranian healthcare system. In this qualitative systematic review, we followed the instructions of the Joanna Briggs Institute (JBI).^35^ According to this instruction, the inclusion and exclusion criteria are:

###  Participants (Population)

 Inclusion criteria relating to participants are not applicable in this systematic review.

###  Phenomena of Interest

 In this study, we considered and included the studies that described challenges, barriers, and difficulties in healthcare policies in Iran.

###  Context

 The Iranian health care setting was considered as the context in this study.

###  Study Types

 In the current systematic review, we considered and included only qualitative studies with all methodological approaches including phenomenology, ethnography, grounded theory, and qualitative sections of mixed method studies.

###  Search Strategy

 Only published studies were considered for inclusion in this research. First, we searched MEDLINE to find the related keywords which included policy, leadership, governance, and Iran, and develop a search strategy. Secondly, databases of MEDLINE, Embase, PubMed, CINAHL, PsycInfo, Scopus, Web of Knowledge, and the Cochrane Library were searched using the main keywords. Persian databases were then searched for articles in the Persian language. In addition, we searched Google Scholar for any possible studies. Also, we screened references of all included studies. No time limitation was considered in the search of this study.

###  Selection of Studies

 After the search was complete, all identified studies were uploaded into the Endnote X8 software, and duplicates were removed. Studies were first screened by the titles and abstracts by two independent reviewers. Then, the full-texts of the included studies were assessed.

###  Quality Appraisal 

 The included studies were appraised for their quality by two independent reviewers using the JBI quality appraisal checklist for qualitative studies.^35^ Any disagreements were resolved through discussion. Studies with a quality appraisal score of seven and above were considered as high-quality.

###  Data Extraction

 Based on the data extraction tools from JBI,^35^ the following data were extracted: citation, publication year, qualitative methodology, study setting, participants, and methods for data collection and analysis. Additionally, we extracted policies, challenges, barriers and difficulties mentioned in the included studies.

###  Data Synthesis

 For data synthesis, we used thematic analysis and synthesis developed by Tomas and Harden.^36^ This approach consists of three stages. First, we read and re-read the primary study findings to structurally code the text line by line according to their meaning and content. In the second stage we reviewed these codes for their similarities and differences and started to inductively group them into a hierarchical tree structure. This process resulted in generating the key descriptive themes distinguished for identified challenges. In the third stage, we went beyond the primary study findings and generated additional concepts or meanings naming ‘analytical themes’. This stage is more dependent on the reviewers’ judgment and insight. We inferred challenges of leadership and governance policies in the Iranian healthcare system. We categorized themes in this stage based on the four functions of the health system (stewardship, financing, resource generation and service delivery).^4^ Two of the reviewers developed the codes and themes independently and then all the reviewers verified and accepted these themes. Through this discussion, challenges and barriers were examined in light of the analytical themes and changes were made. The process was continued until the new analytical themes were sufficiently meaningful to describe all descriptive themes and our inferred challenges.

## Results

###  Study Selection

**Figure 1:**
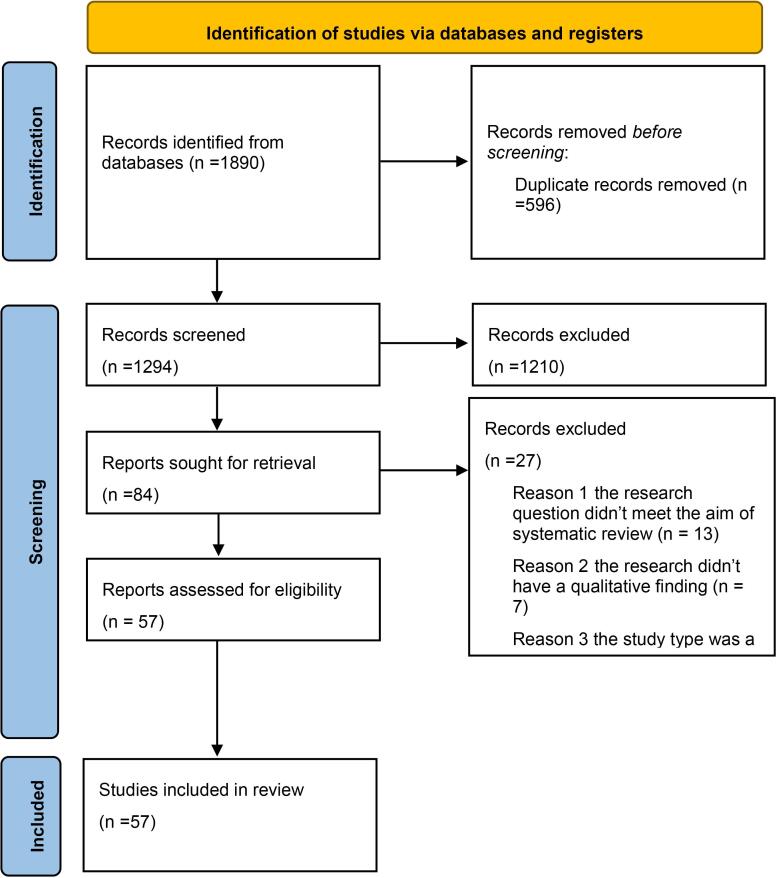
PRISMA Flow Diagram

 The primary search in the databases yielded 1890 records. After screening by title and abstract, 152 articles remained. Finally, 57 papers were included in this systematic review. The reasons for exclusion after full-text review were: (1) the research did not include a governance/ leadership-related policy and (2) the study type was a review or letter to editor. Figure 1 shows the PRISMA flow diagram.

###  Main Characteristics of the Included Studies

 The publication dates of the included studies were between 2010 and 2021, all providing challenges and weaknesses of leadership and governance-related health policies in Iran. The full characteristics of the included studies are indicated in Table S1 (see Supplementary file 1).

###  Methodological Quality

Table 1 summarizes the methodological quality of all the included studies. Most of the included studies had a score of seven and above, and were thus considered high-quality.

**Table 1:** Results of Quality Appraisal of Included Studies

**Citation details**	**Q1: Philosophical Perspective Conjures with the Research Methodology**	**Q2: Research Methodology Conjures with the Objectives **	**Q3: Research Methodology Conjures with the Data Collection Methods **	**Q4: Research Methodology Conjures with the Data Analysis **	**Q5: Research Methodology Conjures with the Interpretation of Results**	**Q6: Statement Locating the Researcher Culturally or Theoretically**	**Q7: Addressing Influence of the Researcher on the Research, and vice-versa**	**Q8: Representation of Participants, and Their Voices**	**Q9: Research Is Ethical**	**Q10: Conclusions appear to flow from the analysis or interpretation of the data**
Afzali et al, 2011^33^	Yes	Yes	Yes	Yes	Yes	Yes	Unclear	Yes	Yes	Yes
Ahmady et al, 2020^37^	Yes	Yes	Yes	Yes	Yes	Unclear	Unclear	Yes	Yes	Yes
Yazdi-Feyzabadi et al, 2015^38^	Yes	Yes	Yes	Yes	Yes	Unclear	Unclear	Yes	Yes	Yes
Bazyar et al, 2020^39^	Yes	Yes	Yes	Yes	Yes	Unclear	Unclear	Unclear	Unclear	Yes
Bazyar et al, 2018^40^	Yes	Yes	Yes	Yes	Yes	Unclear	Unclear	Yes	Yes	Yes
Damari et al, 2013^41^	Yes	Yes	Yes	Yes	Yes	NA	NA	NA	Yes	Yes
Davari et al, 2012^42^	Yes	Yes	Yes	Yes	Yes	NA	NA	NA	Yes	Yes
Doshmangir et al, 2017^43^	Yes	Yes	Yes	Yes	Yes	Unclear	Unclear	Yes	Yes	Yes
Doshmangir et al, 2015^44^	Yes	Yes	Yes	Yes	Yes	Unclear	Unclear	Yes	Yes	Yes
Doshmangir et al, 2016^45^	Yes	Yes	Yes	Yes	Yes	Unclear	Unclear	Yes	Yes	Yes
Doshmangir et al, 2020^46^	Yes	Yes	Yes	Yes	Yes	Unclear	Unclear	Yes	Yes	Yes
Doshmangir et al, 2015^47^	Yes	Yes	Yes	Yes	Yes	Unclear	Unclear	Yes	Yes	Yes
Doshmangir et al, 2018^48^	Yes	Yes	Yes	Yes	Yes	Unclear	Yes	Yes	Yes	Yes
Esmaeili et al, 2015^18^	Yes	Yes	Yes	Yes	Yes	Unclear	Yes	Yes	Yes	Yes
Fardid et al, 2019^49^	Yes	Yes	Yes	Yes	Yes	Unclear	Yes	Yes	Yes	Yes
Gorji et al, 2018^50^	Yes	Yes	Yes	Yes	Yes	Unclear	Unclear	Yes	Yes	Yes
Hassani et al, 2013^51^	Yes	Yes	Yes	Yes	Yes	Unclear	Unclear	Unclear	Yes	Yes
Heydari et al, 2018^52^	Yes	Yes	Yes	Yes	Yes	Unclear	Unclear	Unclear	Yes	Yes
Heydari et al, 2017^53^	Yes	Yes	Yes	Yes	Yes	Unclear	Unclear	Unclear	Yes	Yes
Ibrahimipour et al, 2011^54^	Yes	Yes	Yes	Yes	Yes	Unclear	Unclear	Yes	Yes	Yes
Jafari et al, 2018^55^	Yes	Yes	Yes	Yes	Yes	Unclear	Unclear	Yes	Unclear	Yes
Jafari et al, 2011^56^	Yes	Yes	Yes	Yes	Yes	Unclear	Unclear	Yes	Yes	Yes
Kiani et al, 2021^57^	Yes	Yes	Yes	Yes	Yes	Unclear	Unclear	Yes	Yes	Yes
Markazi-Moghaddam et al, 2014^58^	Yes	Yes	Yes	Yes	Yes	Unclear	Unclear	Yes	Yes	Yes
Mehrolhassani et al, 2013^59^	Yes	Yes	Yes	Yes	Yes	Yes	Unclear	Yes	Unclear	Yes
Mohsenpour et al, 2017^60^	Yes	Yes	Yes	Yes	Yes	Unclear	Unclear	Yes	Yes	Yes
Naghdi et al, 2017^61^	Yes	Yes	Yes	Yes	Yes	Yes	Unclear	Yes	Yes	Yes
Naseriasl et al, 2018^62^	Yes	Yes	Yes	Yes	Yes	Unclear	Yes	Yes	Yes	Yes
Nekoei-Mogadam et al, 2018^63^	Yes	Yes	Yes	Yes	Yes	Yes	Unclear	Yes	Yes	Yes
Nekoei- Mogadam et al, 2013^64^	Yes	Yes	Yes	Yes	Yes	Unclear	Unclear	Yes	Yes	Yes
Parsa et al, 2015^65^	Yes	Yes	Yes	Yes	Yes	Yes	Unclear	Yes	Yes	Yes
Pourabbasi et al, 2019^66^	Yes	Yes	Yes	Yes	Yes	Unclear	Unclear	Yes	Yes	Yes
Poursheikhali, 2021^67^	Yes	Yes	Yes	Yes	Yes	Unclear	Unclear	Yes	Yes	Yes
Ravaghi et al, 2014 ^68^	Yes	Yes	Yes	Yes	Yes	Unclear	Unclear	Yes	Yes	Yes
Rooddehghan et al, 2014^69^	Yes	Yes	Yes	Yes	Yes	Unclear	Unclear	Yes	Yes	Yes
Sabet et al, 2017^70^	Yes	Yes	Yes	Yes	Yes	Unclear	Unclear	Yes	Yes	Yes
Sadeghi et al, 2016^71^	Yes	Yes	Yes	Yes	Yes	Unclear	Unclear	Yes	Yes	Yes
Sajadi et al, 2016^72^	Yes	Yes	Yes	Yes	Yes	Unclear	Unclear	Unclear	Yes	Yes
Sajadi et al, 2014^73^	Yes	Yes	Yes	Yes	Yes	Unclear	Unclear	Yes	Yes	Yes
Seyedin et al, 2021^74^	Yes	Yes	Yes	Yes	Yes	Unclear	Unclear	Yes	Yes	Yes
Tabrizi et al, 2021^75^	Yes	Yes	Yes	Yes	Yes	Unclear	Unclear	NA	Yes	Yes
Yaghoubian et al, 2019^76^	Yes	Yes	Yes	Yes	Yes	Unclear	Unclear	Yes	Yes	Yes
Zalani et al, 2018^77^	Yes	Yes	Yes	Yes	Yes	Unclear	Unclear	Unclear	Yes	Yes
Abedi et al, 2017^78^	Yes	Yes	Yes	Yes	Yes	Unclear	Unclear	Yes	Yes	Yes
Abolhallaje et al, 2016^79^	Yes	Yes	Yes	Yes	Yes	Unclear	Unclear	NA	Unclear	Yes
Anjomshoa et al2021^27^	Yes	Yes	Yes	Yes	NA	Unclear	Unclear	NA	Yes	Yes
Doshmangir et al, 2019^80^	Yes	Yes	Yes	Yes	Yes	Unclear	Unclear	Yes	Yes	Yes
Falahat et al, 2013^81^	Yes	Yes	Yes	Yes	Yes	Unclear	Unclear	NA	Yes	Yes
Farzadfar et al, 2017^82^	Yes	Yes	Yes	Yes	Yes	Unclear	Unclear	Yes	Yes	Yes
Khankeh et al, 2020^83^	Yes	Yes	Yes	Yes	Yes	Yes	Yes	Yes	Yes	Yes
Marnani, 2010^84^	Yes	Yes	Yes	Yes	Yes	NA	NA	Yes	NA	Yes
Mohammadpour et al, 2020^85^	Yes	Yes	Yes	Yes	Yes	Unclear	Yes	Yes	Yes	Yes
Moshiri et al, 2016^86^	Yes	Yes	Yes	Yes	Yes	Unclear	Yes	Yes	Yes	Yes
Nejatzadegan et al, 2016^87^	Yes	Yes	Yes	Yes	Yes	NA	Yes	Yes	Yes	Yes
Safizadehe et al, 2016^88^	Yes	Yes	Yes	Yes	Yes	NA	Yes	Yes	Yes	Yes
Sarvestani et al, 2017^70^	Yes	Yes	Yes	Yes	Yes	Unclear	Yes	Yes	Yes	Yes
Takian et al, 2011^89^	Yes	Yes	Yes	Yes	Yes	NA	Yes	Yes	Yes	Yes

###  Main Findings

 The process of thematic synthesis yielded 157 structured codes from the primary studies and among the four analytical themes of health system functions, which were determined prior to the analysis, 11 main descriptive themes of challenges in leadership and governance-related health policies (See Table 2). The main descriptive themes included development and implementation of hospital autonomy policy, challenges to the whole health system, medical university’s governance: board of trustees, healthcare payment system, reaching sustainable universal health insurance coverage, informal payments, insurance system, induced demand, implementation of strategic purchasing of health services in Iran, family physician program, purchaser–provider split in the implementation of family physician and rural health insurance in Iran, and PHC human resources.

**Table 2:** Summary of Thematic Synthesis Process

**Analytical Theme (Health System Functions)**	**Main Descriptive Themes**	**Challenges (Codes in the Text)**
Stewardship	Policy of hospital autonomy	Shortage of piloting studies Implementation of autonomy policy is not evidence-based Lack of assessing feasibility of the policy Weak collaboration among related stakeholders Lack of legal frameworks Culture-related challenges issues in healthcare system Misunderstanding in the interpretation of the autonomy policy Top-down managerial approach Lack of financing resources Hasty implementation
	Systemic Dysfunctions in Healthcare Management and Planning	Technical bankruptcy of the health care system Unresponsive healthcare system Politicization of the healthcare system Dysfunctional and short-term decision making Low priority given to the health care system Not an excellence-based recruitment of managers Assignments of management in the short period of time Political challenges in replacing managers Passive planning Piecemeal planning Non-localized plans Incoherent leadership Incomplete referral system Separation between public and private sectors
	Medical University's Governance: Board of trustees	Organizational ignorance inside and outside of universities Lack of awareness board’s mission and vision Lack of awareness of regulatory organizations about the board’s position Inability of new universities Lack of knowledge among some of the members about rules and regulations
Financing	Healthcare Payment System	No income ceiling for physicians Lack of consistency between different payment systems Lack of proper linkage between officials Lack of guidelines
	Reaching sustainable universal health insurance coverage	Undefined coverage rate Coverage overlap among population Role of the government Leakage in supportive subsidies Providers that are not conscious about costs High managerial costs Lack of evidence-based and adapted clinical practice guidelines Lack of a health technology assessment system Commitment and responsibility of government Not sustainable management positions Requirements for rules and constitutions
	Informal payments	Lack of people awareness of their rights as a reason for informal payments Insufficiency of law and the complaints process as a reason for informal payments Unrealistic, insufficient, and inequitable tariﬀs Lack of sufficient monitoring laws High discrimination among healthcare providers
	Iranian insurance system	Ill-defined status of insurance companies Unclear approach of insurance system Unclear insurance coverage Non-comprehensive rules despite being many in numbers Centralized policy making and management Overlapping of insurance coverage Unclear and unjustified policies of financial support Conflict of interest in financing Challenges related to complementary insurance Using high-technology based and high-cost drugs without considering cost-effectiveness issues Unclear coordination and continuity among levels of health care system Shortage of an integrated health system Lack of policy in the supply side entry Non-real tariffs Lack of coordinated payment mechanism
	Induced demand	Educational role of health system Lack of development in evidence-based policymaking Lack of much oversight on technologies that are imported Lack of enough rules about insurance system Lack of private insurance system Weak coordination between public and private sector
	Strategic purchasing of healthcare services	Lack of enforcing rules and laws Lack of knowledge among stakeholders Lack of appropriate criteria about required information Lack of appropriate planning for strategic purchasing Effect of purchaser goals on priority settings of strategic purchasing Weaknesses in decision-making system and evidence-informed policymaking Conflicting interest among different purchasers Lack of hearing patients voice for purchasing health services Lack of appropriate monitoring mechanism for purchasers Low engagement rate of policy making associations Less clarity in role definition of nongovernmental organizations (NGOs) Less clarity in supervision rules for non-public sector Instable management Shortage of trust in insurance organizations Conflict of interest among top managers of ministry of health Lack of rules and regulations Shortage of provider and purchaser split (PPS) Lack of clear mission Lack of appropriate structure and functions Less adherence to clinical practice guidelines and protocols Lack of evidence-based and adapted clinical practice guidelines and protocols Lack of clarity in quality, efficiency, effectiveness, and safety indicators Lack of consistence among 3 dimensions of universal health coverage (group, services and cost) for vulnerable population Low accessibility, affordability, availability and comprehensiveness among healthcare services Paying less attention to components of market Lack of competition among healthcare providers Inconsistency among policy-making authorities Lack of macroeconomic currency policies in in country Lack of compatibility of universities’ structures with responsibilities HIS challenges in PHC based: Governing bureaucracy principle on the local health systems Poor transparency of performance and duties of end users Cultural issues in expressing the functional facts and errors The lack of guidelines in order to record data equally among areas Poor use of information in the local decision making Poor intersectional collaboration and communication in urban areas
Service delivery	Purchaser–provider split in family physician policy	Lack of acculturation as the major weakness of this program Lack of awareness of people about the goals of the program and their assumption to see a family physician only for illness Abrupt start as the major weakness of this program Failure in expertizing, managing and supervising the plan as challenges of this new program Lack of proper planning of the payment and referral systems Lack of training physicians about families, their needs, follow-ups, and communication Lack of orientation of medical specialists with this program This program as a good source of income for medical specialists An increase in expenses despite of goal of the program that was decreasing or managing the referrals to medical specialists Denigration of family physicians and their dropped social position and their decreased self-esteem and People's distrust of them Evaluation and monitoring by two organizations Lack of compatibility between physician’s and controllers’ level of education Lack of valid and reliable checklists Lack of monitoring among family physician team members Lack of supportive environment Lack of comprehensiveness Change in the program’s nature during practice Ignoring the physician’s position Weakness of infrastructures in residential welfare and transportation Lack of training for health care providers Not welcoming approach among physicians Poor infrastructure for urban family physician program Egoistic manner of medical specialist Poor incentive mechanism Denigration of family physicians Law deviation Discrimination Resistance against implementation Lack of stable managerial positions in the Supreme Council of Insurance Conflict of interest among members of the Supreme Council of Insurance Lack of clarity in path of the cabinet Unification and promotion of laws
		Economic Crisis Low Customer Satisfaction Political Instability Chaotic Healthcare System Lack of appropriate management of the Healthcare System Fragmented Healthcare System
Resource generation	PHC human resources	Vacant posts at PHC centers Need to retrain new workers Weakness in upper levels of health systems Lack of PHC-centered approach in university education Lack of PHC-centered approach in training practitioners Lack of PHC-related issues among research priorities of universities Lack of clarity in evaluation of PHC staff Lack of PHC-related approach in curriculum of health services management students Less opportunity of employing health services management graduates in the PHC system Failure in training of health services managers

## Discussion

 We conducted this systematic review to summarize the challenges and weaknesses of leadership and governance-related health policies in Iran. Challenges and weakness were categorized based on the four functions of the health system (stewardship, financing, resource generation and service delivery). The main descriptive themes included the hospital autonomy policy, challenges to the entire health system, governance of medical universities, healthcare payment systems, sustainable universal health insurance coverage, informal payments, insurance systems, induced demand, strategic purchasing of health services, the family physician program, family physician and rural health insurance programs, and primary healthcare human resources.

 A key goal of the Sustainable Development Goals (SDGs) is to ensure that everyone can access the health services they require without facing financial difficulties. This is called universal health coverage (UHC) and it also supports the achievement of other health-related SDGs.^90^ Iran has faced several political challenges in recent years, such as sanctions, protests, and regional tensions that may affect its health system performance and UHC goals.^91^ The findings of this study also indicate that financial challenges dominate the health system, which can be one of the obstacles to achieving UHC goals. The Iranian government funds its healthcare system mainly through three sources: general government budget, health insurance, and out-of-pocket payments. The general taxation revenue is allocated to the Ministry of Health and Medical Education (MOHME), which is the main stewardship of public health services provision and subsidizing medical education and research.^92^ The health insurance revenue comes from various schemes that cover different segments of the population, such as the Social Security Organization, the Medical Service Insurance Organization, the Military Personnel Insurance Organization, and the Emdad-e-Emam Committee. The out-of-pocket payments are made by the patients directly to the health care providers, especially in the private sector.^93^ Consistent with our results, other studies have shown that despite the recent efforts to expand health insurance coverage since the fifth national development plan, some groups of people in Iran are still not covered by any scheme nor receive inadequate benefits.^94,95^ The private medical tariffs have risen tenfold since 2003, when the Iranian Medical Council established them for this sector. The public health sector and the related health insurance organizations could not keep up with this increase, which led to a decrease in the health service’s access and an increase in out-of-pocket payments in the private sector.^96^ Davari and co-authors in their study argued that these challenges require urgent and comprehensive reforms to improve the performance and sustainability of the health insurance system. They also pointed out lack of coordination, integration, and accountability among the different actors, which leads to duplication, inefficiency, and inequity in the provision and financing of health services.^42^

 According to results of our study, informal payment is a major challenge in the Iranian health system, and it can have negative consequences for both the health sector resources and the financial burden of patients. Some of the factors that contribute to high informal or out-of-pocket payment in Iran, such as unfair tariff valuation of health services,^34^ lack of a clear definition of the basic benefit package, fragmentation of health insurance schemes.^42,97^ and low level of risk pooling.^98^ Iran’s health system can improve its responsiveness by increasing and managing the health expenditure per capita, which is the lowest compared to countries such as Australia, Luxembourg, Qatar and United States.^99^ There are other possible strategies to overcome these challenges, such as expanding the coverage of the vulnerable groups,^39^ developing a national health accounts system,^93^ strengthening the regulatory framework, and enhancing public awareness and participation,^100^ and adopting a strategic purchasing approach.^39^ Health services are bought by both public and private actors in countries such as Iran. Among the purchasers of health services are health ministries and insurance organizations. This is contrary to the WHO’s recommendation to have one stewardship for purchasing health services, as there are various stewardships in this situation.^101^ Having a single stewardship can help increase the transparency, accountability, and efficiency of health financing, as well as reduce the duplication and fragmentation of health services. However, achieving a single stewardship is not easy, and requires strong political commitment and leadership, as well as institutional capacity and governance.^102^

 The health systems in some developed countries, such as Japan, Singapore, and the Republic of Korea, also need to deal with the growing challenges of maintaining their financial sustainability.^103^ Some of the above mentioned countries have implemented different types of reforms related to health financing to cope with these challenges, such as expanding pre-paid financing mechanisms, improving strategic purchasing, strengthening domestic financing, and enhancing priority setting and benefits design processes.^104^ However, the effects of these reforms on health outcomes and health equity are not well understood and require further evaluation.^105^

 One of the other challenges that emerged from the results of this study is induced demand in health services. Studies show that going to the doctor for simple issues and asking for too many services can create induced demand, especially if the patients have too much trust in the doctor and do not question their decisions.^106^ However, patients have limited medical information and choosing many services will not necessarily promote their well-being.^107^ In fact, research has shown that overuse of health care services can lead to harm, waste, and dissatisfaction among patients ^108,109^. Therefore, it is important for patients to be informed and empowered to make rational and appropriate decisions about their health care needs.^110^ However, there are also other factors that influence induced demand, such as the payment system, the referral system, the supervision and monitoring system, and the cultural and social factors.^111^ These factors affect both the supply and demand of health care services, and they need comprehensive reforms and policies.^112^ Some possible reforms and policies are paying providers a fixed amount per person, not per service; making primary care physicians the first contact for patients; evaluating and auditing providers based on performance, quality, and standards; and supporting the factors that help patients and providers use health care services rationally and appropriately.^113^

 Another factor is the overemphasis on the treatment, rather than preventive and primary care, which may result in inefficiencies, high costs, and low quality of care.^114^ Existence of a referral system is a key element in success of the family physician program that ensures suitable and economically accessible health care services for the population.^115^ However, according to the results of this study, Iran does not have a clear and effective referral system that can direct patients to the appropriate level of care based on their needs. This may lead to unnecessary use of specialized and tertiary care, as well as dissatisfaction among patients and providers.^116^ The Referral Systems Assessment and Monitoring Toolkit suggests two main components for evaluating how well a referral system works: referral system assessment (RSA) and referral system monitoring (RSM). RSA involves collecting and analyzing different types of data to examine the referral system at a specific point in time. RSM involves measuring and tracking the referral system over time using indicators and tools. These components can help assess the referral system in terms of its relevance, efficiency, effectiveness, impact, and sustainability. They can also help compare the referral system with its goals and expectations, as well as with other systems.^116^

## Strengths of the Study

 The study employed a comprehensive search strategy across multiple databases, ensuring a wide coverage of relevant literature. The inclusion criteria were clearly defined, allowing for a focused and targeted selection of studies that align with the research question. The use of standardized appraisal checklists for methodological quality assessment, as well as the involvement of independent reviewers, enhances the rigor of the study’s quality assessment process. The use of a standardized data extraction tool ensures a systematic and consistent approach to extracting relevant data from the included papers. The study employed a robust thematic analysis and synthesis approach, involving multiple stages and independent reviewers, which enhances the credibility and dependability of the findings.

## Limitations of the Study

 The focus on published studies may introduce publication bias, as relevant unpublished or grey literature may not have been included in the review. While the use of standardized appraisal checklists is a strength, the limitations and subjectivity of these tools should be acknowledged. The process of generating analytical themes in the data synthesis stage may be influenced by the reviewers’ judgment and insight, potentially introducing subjectivity in the interpretation of the findings. The findings may be specific to the context of the Iranian healthcare system and may not be directly generalizable to other healthcare systems without considering contextual differences.

## Conclusion

 This comprehensive analysis of the challenges and weaknesses of leadership and governance-related health policies in Iran revealed numerous challenges and obstacles that need to be addressed to improve the overall performance of the healthcare system. These challenges encompass various aspects of healthcare governance, financing, service delivery, and resource generation. From the lack of pilot studies and formal assessments for policy implementation to the complex issues related to healthcare payment systems and strategic purchasing of health services, the findings underscore the multifaceted nature of the challenges faced by the healthcare system.

 The identified challenges range from technical bankruptcy, unresponsive healthcare systems, and politicization of the healthcare system to issues related to decision-making, priority setting, and leadership. Furthermore, the findings shed light on the inadequacies in the governance structures of medical universities, financing mechanisms, and the healthcare insurance system. The challenges also extend to the implementation of programs such as the family physician program, with issues related to acculturation, awareness, planning, and performance evaluation.

 Moreover, the analysis highlights the broader contextual challenges, including economic crises, low customer satisfaction, political instability, and mismanagement of the healthcare system. These challenges collectively contribute to a chaotic and fragmented healthcare system, impacting resource generation and the availability of skilled human resources at primary healthcare centers.

 Addressing these challenges will require a comprehensive and multi-faceted approach, encompassing reforms in policy-making, governance structures, financing mechanisms, service delivery models, and human resource management. It is imperative to develop and implement evidence-based policies, improve coordination among stakeholders, enhance transparency, and strengthen the capacity of the healthcare workforce. Furthermore, efforts to address the systemic challenges should be accompanied by a focus on improving public awareness, enhancing regulatory frameworks, and fostering collaboration between the public and private sectors.

 In conclusion, the identified challenges underscore the urgent need for strategic reforms and interventions to overcome the complex issues plaguing the healthcare system. By addressing these challenges, policymakers and top healthcare managers might ensure that the population have access to high-quality care in a more responsive healthcare system.

## Supplementary Files

Supplementary file 1 contains Table S1.

## References

[1] Almaspoor Khangah H, Jannati A, Imani A, Salimlar S, Derakhshani N, Raef B (2017). Comparing the health care system of Iran with various countries. Health Scope.

[2] Harirchi I, Hajiaghajani M, Sayari A, Dinarvand R, Sajadi HS, Mahdavi M (2020). How health transformation plan was designed and implemented in the Islamic Republic of Iran?. Int J Prev Med.

[3] Han W (2012). Health care system reforms in developing countries. J Public Health Res.

[4] World Health Organization (WHO). The World Health Report 2000: Health Systems: Improving Performance. WHO; 2000.

[5] Stokes J, Gurol-Urganci I, Hone T, Atun R (2015). Effect of health system reforms in Turkey on user satisfaction. J Glob Health.

[6] Akinci F, Mollahaliloğlu S, Gürsöz H, Oğücü F (2012). Assessment of the Turkish health care system reforms: a stakeholder analysis. Health Policy.

[7] Galárraga O, Sosa-Rubí SG, Salinas-Rodríguez A, Sesma-Vázquez S (2010). Health insurance for the poor: impact on catastrophic and out-of-pocket health expenditures in Mexico. Eur J Health Econ.

[8] Laurell AC (2007). Health system reform in Mexico: a critical review. Int J Health Serv.

[9] Towse A, Mills A, Tangcharoensathien V (2004). Learning from Thailand’s health reforms. BMJ.

[10] Jafari M, Ghasemyani S, Khodayari-Zaranq R, Raoofi S (2021). Health transformation plan achievements and outcomes in Iran (2014-2020): a scoping review. Med J Islam Repub Iran.

[11] Doshmangir L, Rashidian A, Jafari M, Takian A, Ravaghi H (2015). Opening the black box: the experiences and lessons from the public hospitals autonomy policy in Iran. Arch Iran Med.

[12] Abdi Z, Majdzadeh R, Ahmadnezhad E (2019). Developing a framework for the monitoring and evaluation of the Health Transformation Plan in the Islamic Republic of Iran: lessons learned. East Mediterr Health J.

[13] Bagheri Lankarani K, Alavian SM, Peymani P (2013). Health in the Islamic Republic of Iran, challenges and progresses. Med J Islam Repub Iran.

[14] Malekafzali H (2009). Primary health care in the rural area of the Islamic Republic of Iran. Iran J Public Health.

[15] Manenti A (2011). Health situation in Iran. Med J Islam Repub Iran.

[16] Shadpour K (2000). Primary health care networks in the Islamic Republic of Iran. East Mediterr Health J.

[17] Jabbari H, Tabibi J, Delgoshaii B, Mahmoudi M, Bakhshian F. Comparative study of decentralization mechanisms in health care delivery in different countries and suggesting a model for Iran. Health Management Journal 2007;10(27):33-40. [Persian].

[18] Esmaeili R, Hadian M, Rashidian A, Shariati M, Ghaderi H (2014). Family medicine in Iran: facing the health system challenges. Glob J Health Sci.

[19] Letafat M, Beyranvand T, Aryankhesal A, Behzadifar M, Behzadifar M (2018). Universal health coverage (UHC) in Iran. Iran J Public Health.

[20] Vosoogh Moghaddam A, Damari B, Alikhani S, Salarianzedeh M, Rostamigooran N, Delavari A (2013). Health in the 5th 5-years Development Plan of Iran: main challenges, general policies and strategies. Iran J Public Health.

[21] Moradi-Lakeh M, Vosoogh Moghaddam A (2015). Health sector evolution plan in Iran; equity and sustainability concerns. Int J Health Policy Manag.

[22] Heshmati B, Joulaei H (2016). Iran’s health-care system in transition. Lancet.

[23] Olyaeemanesh A, Behzadifar M, Mousavinejhad N, Behzadifar M, Heydarvand S, Azari S (2018). Iran’s health system transformation plan: a SWOT analysis. Med J Islam Repub Iran.

[24] Sajadi HS, Majdzadeh R (2019). From primary health care to universal health coverage in the Islamic Republic of Iran: A Journey of Four Decades. Arch Iran Med.

[25] Takian A, Doshmangir L, Rashidian A (2013). Implementing family physician programme in rural Iran: exploring the role of an existing primary health care network. Fam Pract.

[26] Ghasemyani S, Raoofi S, Hamidi H, Khodayari-Zarnaq R (2022). Iran’s health transformation plan; main issues and opportunities for improvement: a systematic review. Iran J Public Health.

[27] Anjomshoa M, Akbari Sari A, Takian A (2021). Assessing progress in the national health financing system towards universal health coverage in Iran: a mixed-method study protocol. Health Res Policy Syst.

[28] Bordbar S, Gholampoor H, Jalali FS, Delavari S (2023). The Effect of Iran Health Transformation Plan on Equity in Health Financing: A Systematic Review. Iran J Public Health.

[29] Mousavi SM, Sadeghifar J (2016). Universal health coverage in Iran. Lancet Glob Health.

[30] Ahmadnezhad E, Murphy A, Alvandi R, Abdi Z (2019). The impact of health reform in Iran on catastrophic health expenditures: equity and policy implications. Int J Health Plann Manage.

[31] Soltani S, Rezaei S, Kazemi-Karyani A, Azimi J, Jalili F, Roshani B (2023). The effect of Iran’s health sector evolution plan on hospitals performance indicators: an interrupted time series analysis. Front Health Serv.

[32] Saran M, Teli BD, Rezapour A, Motlagh SN, Behzadifar M, Haghighatfard P (2023). The impact of the Iranian health transformation plan policy on equitable access to medical imaging services in West Iran. BMC Research Notes.

[33] Afzali HH, Moss JR, Mahmood MA (2011). Exploring health professionals’ perspectives on factors affecting Iranian hospital efficiency and suggestions for improvement. Int J Health Plann Manage.

[34] Meskarpour Amiri M, Teymourzadeh E, Ravangard R, Bahadori M. Health informal payments and their main determinants: the case of Iran. Proceedings of Singapore Healthcare. 2019:2010105818822594. 10.1177/2010105818822594.

[35] Joanna Briggs Institute (JBI). Joanna Briggs Institute Reviewers’ Manual: 2014 Edition. Australia: JBI; 2014. p. 88-91.

[36] Thomas J, Harden A (2008). Methods for the thematic synthesis of qualitative research in systematic reviews. BMC Med Res Methodol.

[37] Ahmady S, Khajeali N, Mirmoghtadaie Z (2020). Challenges and opportunities of acquiring scientific authority in medical sciences: determination of the experts’ views based on qualitative content analysis. J Adv Med Educ Prof.

[38] Yazdi-Feyzabadi V, Emami M, Mehrolhassani MH (2015). Health information system in primary health care: the challenges and barriers from local providers’ perspective of an area in Iran. Int J Prev Med.

[39] Bazyar M, Rashidian A, Kane S, Vaez Mahdavi MR, Akbari Sari A, Doshmangir L (2016). Policy options to reduce fragmentation in the pooling of health insurance funds in Iran. Int J Health Policy Manag.

[40] Bazyar M, Rashidian A, Alipouri Sakha M, Doshmangir L, Rahimi N, Ranjbar M (2019). Stakeholders analysis of merging social health insurance funds in Iran: what kind of interests they may gain or lose?. Int J Health Plann Manage.

[41] Damari B, Aminloo H, Farzan H, Rahbari M, Alikhani S (2013). Ways to improve the current performance of the boards of trustees of medical universities in Iran. Iran J Public Health.

[42] Davari M, Haycox A, Walley T (2012). The Iranian health insurance system; past experiences, present challenges and future strategies. Iran J Public Health.

[43] Doshmangir L, Bazyar M, Doshmangir P, Mostafavi H, Takian A (2017). Infrastructures required for the expansion of family physician program to urban settings in Iran. Arch Iran Med.

[44] Doshmangir L, Doshmangir P, Abolhassani N, Moshiri E, Jafari M (2015). Effects of targeted subsidies policy on health behavior in Iranian households: a qualitative study. Iran J Public Health.

[45] Doshmangir L, Rashidian A, Jafari M, Ravaghi H, Takian A (2016). Fail to prepare and you can prepare to fail: the experience of financing path changes in teaching hospitals in Iran. BMC Health Serv Res.

[46] Doshmangir L, Rashidian A, Kouhi F, Gordeev VS (2020). Setting health care services tariffs in Iran: half a century quest for a window of opportunity. Int J Equity Health.

[47] Doshmangir L, Rashidian A, Ravaghi H, Takian A, Jafari M (2015). The experience of implementing the board of trustees’ policy in teaching hospitals in Iran: an example of health system decentralization. Int J Health Policy Manag.

[48] Doshmangir L, Rashidian A, Takian A, Doshmangir P, Mostafavi H (2018). Payment system of urban family physician programme in the Islamic Republic of Iran: is it appropriate?. East Mediterr Health J.

[49] Fardid M, Jafari M, Vosoogh Moghaddam A, Ravaghi H (2019). Challenges and strengths of implementing urban family physician program in Fars province, Iran. J Educ Health Promot.

[50] Abolghasem Gorji H, Mousavi S, Shojaei A, Keshavarzi A, Zare H (2018). The challenges of strategic purchasing of healthcare services in Iran Health Insurance Organization: a qualitative study. Electron Physician.

[51] Hassani SA, Mobaraki H, Bayat M, Mafimoradi S (2013). Right place of human resource management in the reform of health sector. Iran J Public Health.

[52] Heydari M, Seyedin H, Jafari M, Dehnavieh R (2018). Stakeholder analysis of Iran’s health insurance system. J Educ Health Promot.

[53] Heydari MR, Kalateh Sadati A, Bagheri Lankarani K, Imanieh MH, Baghi H, Lolia MJ (2017). The evaluation of urban community health centers in relation to family physician and primary health care in southern Iran. Iran J Public Health.

[54] Ibrahimipour H, Maleki MR, Brown R, Gohari M, Karimi I, Dehnavieh R (2011). A qualitative study of the difficulties in reaching sustainable universal health insurance coverage in Iran. Health Policy Plan.

[55] Jafari M, Habibirad A, Pourtaleb A, Salarianzadeh MH (2018). Health system organizational reform in governing Iranian public hospitals: a content analysis to comprehend the barriers in Board of Trustees’ hospitals. Int J Health Plann Manage.

[56] Jafari M, Rashidian A, Abolhasani F, Mohammad K, Yazdani S, Parkerton P (2011). Space or no space for managing public hospitals; a qualitative study of hospital autonomy in Iran. Int J Health Plann Manage.

[57] Kiani MM, Khanjankhani K, Takbiri A, Takian A (2021). Refugees and sustainable health development in Iran. Arch Iran Med.

[58] Markazi-Moghaddam N, Aryankhesal A, Arab M (2014). The first stages of liberalization of public hospitals in Iran: establishment of autonomous hospitals and the barriers. Iran J Public Health.

[59] Mehrolhassani MH, Emami M (2013). Change theory for accounting system reform in health sector: a case study of Kerman University of Medical Sciences in Iran. Int J Health Policy Manag.

[60] Mohsenpour SR, Arab M, Emami Razavi SH, Akbari Sari A (2017). Exploring the challenges of the Iranian parliament about passing laws for resource allocation in healthcare: a qualitative study. Electron Physician.

[61] Naghdi S, Moradi T, Tavangar F, Bahrami G, Shahboulaghi M, Ghiasvand H (2017). The barriers to achieve financial protection in Iranian health system: a qualitative study in a developing country. Ethiop J Health Sci.

[62] Naseriasl M, Janati A, Amini A, Adham D (2018). Referral system in rural Iran: improvement proposals. Cad Saude Publica.

[63] Nekoei-Moghadam M, Amiresmaili M, Iranemansh M, Iranmanesh M (2018). Hospital accreditation in Iran: a qualitative case study of Kerman hospitals. Int J Health Plann Manage.

[64] Nekoei-Moghadam M, Esfandiari A, Ramezani F, Amiresmaili M (2013). Informal payments in healthcare: a case study of Kerman province in Iran. Int J Health Policy Manag.

[65] Parsa M, Aramesh K, Nedjat S, Kandi MJ, Larijani B (2015). Informal payments for health care in Iran: results of a qualitative study. Iran J Public Health.

[66] Pourabbasi A, Akbari H, Akhvan AA, Haghdoost AA, Kheiry Z, Dehnavieh R (2019). Analysis of Iran’s national medical education evolution and innovation plan using the Michelle and Scott’s model of policymaking. J Adv Med Educ Prof.

[67] Poursheikhali A, Dehnavieh R (2020). How do primary care providers work together in the Iranian PHC system?. Med J Islam Repub Iran.

[68] Ravaghi H, Rafiei S, Heidarpour P, Mohseni M (2014). Facilitators and barriers to implementing clinical governance: a qualitative study among senior managers in Iran. Iran J Public Health.

[69] Rooddehghan Z, Nikbakht Nasrabadi A, Parsa Yekta Z (2014). Components of equity-oriented health care system: perspective of Iranian nurses. Glob J Health Sci.

[70] Sabet Sarvestani R, Najafi Kalyani M, Alizadeh F, Askari A, Ronaghy H, Bahramali E (2017). Challenges of family physician program in urban areas: a qualitative research. Arch Iran Med.

[71] Sadeghi A, Barati O, Bastani P, Daneshjafari D, Etemadian M (2016). Strategies to develop and promote public-private partnerships (PPPs) in the provision of hospital services in Iran: a qualitative study. Electron Physician.

[72] Sajadi HS, Hadi M (2016). Promoting the medical university’s governance: content analysis of decisions made by the medical university’s governing bodies. Arch Iran Med.

[73] Sajadi HS, Maleki M, Ravaghi H, Farzan H, Aminlou H, Hadi M (2014). Evaluation of board performance in Iran’s universities of medical sciences. Int J Health Policy Manag.

[74] Seyedin H, Afshari M, Isfahani P, Hasanzadeh E, Radinmanesh M, Corani Bahador R (2021). The main factors of supplier-induced demand in health care: a qualitative study. J Educ Health Promot.

[75] Tabrizi JS, Gharibi F (2021). Developing national functional accreditation model for primary healthcares with emphasis on family practice in Iran. Korean J Fam Med.

[76] Yaghoubian S, Mahmoudi G, Jahani MA (2020). The requirements of strategic purchasing of health services for cancer patients: a qualitative study in Iran. Health Care Manag (Frederick).

[77] Salehi Zalani G, Khalilnezhad R, Mirbahaeddin E, Shokri A, Kashkalani T, Bayat M (2018). Human resources for health strategies: the way to achieve universal health coverage in the Islamic Republic of Iran. East Mediterr Health J.

[78] Abedi G, Marvi A, Soltani Kentaie SA, Abedini E, Asadi Aliabadi M, Safizadehe Chamokhtari K, et al. SWOT analysis of implementation of urban family physician plan from the perspective of beneficiaries: a qualitative study. J Mazandaran Univ Med Sci 2017;27(155):79-93. [Persian].

[79] Abolhallaje M, Nazari A, Abdollah Oghli Mirali B, Javani A. Assessing new financial system in medical universities in Iran using SWOT analysis. J Mazandaran Univ Med Sci 2016;26(142):186-93. [Persian].

[80] Doshmangir L, Moshiri E, Mostafavi H, Alipouri Sakha M, Assan A (2019). Policy analysis of the Iranian Health Transformation Plan in primary healthcare. BMC Health Serv Res.

[81] Falahat K, Baradaran Eftekhari M, Malekafzali H, Setareh Forouzan A, Dejman M (2013). Governance in community-based health programmes in IR of Iran. J Pak Med Assoc.

[82] Farzadfar F, Jafari S, Rahmani K, Valiee S, Bidarpour F, Molasheikhi M (2017). Views of managers, health care providers, and clients about problems in implementation of urban family physician program in Iran: a qualitative study. Sci J Kurdistan Univ Med Sci.

[83] Khankeh HR, Bagheri Lankarani K, Zarei N, Joulaei H (2021). Three decades of healthcare system reform in Iran from the perspective of universal health coverage: a macro-qualitative study. Iran J Med Sci.

[84] Barati Marnani A, Teymourzadeh E, Bahadori M, Ravangard R, Saeid Pour J (2012). Challenges of a large health insurance organization in Iran: a qualitative study. Int J Collab Res Intern Med Public Health.

[85] Mohammadpour M, Bastani P, Brennan D, Ghanbarzadegan A, Bahmaei J (2020). Oral health policymaking challenges in Iran: a qualitative approach. BMC Oral Health.

[86] Moshiri E, Rashidian A, Arab M, Khosravi A (2016). Using an analytical framework to explain the formation of primary health care in rural Iran in the 1980s. Arch Iran Med.

[87] Nejatzadegan Z, Ebrahimipour H, Hooshmand E, Tabatabaee SS, Esmaili H, Vafaee Najar A (2016). Challenges in the rural family doctor system in Iran in 2013-14: a qualitative approach. Fam Pract.

[88] Safizadehe Chamokhtari K, Abedi G, Marvi A. Analysis of the patient referral system in urban family physician program, from stakeholders` perspective using SWOT approach: a qualitative study. J Mazandaran Univ Med Sci 2018;28(161):75-87. [Persian].

[89] Takian A, Rashidian A, Kabir MJ (2011). Expediency and coincidence in re-engineering a health system: an interpretive approach to formation of family medicine in Iran. Health Policy Plan.

[90] Gilbert K, Park K, Capuano C, Soakai TS, Slatyer B (2019). Achieving UHC in the Pacific, a closer look at implementation: summary of a report for Pacific health ministers. Health Syst Reform.

[91] Dehghani M, Mesgarpour B, Akhondzadeh S, Azami-Aghdash S, Ferdousi R (2021). How the US sanctions are affecting the health research system in Iran?. Arch Iran Med.

[92] Davari M, Haycox A, Walley T (2012). Health care financing in Iran; is privatization a good solution?. Iran J Public Health.

[93] Zakeri M, Olyaeemanesh A, Zanganeh M, Kazemian M, Rashidian A, Abouhalaj M (2015). The financing of the health system in the Islamic Republic of Iran: a National Health Account (NHA) approach. Med J Islam Repub Iran.

[94] Dehnavieh R, Rahimi H (2017). Basic health insurance package in Iran: revision challenges. Iran J Public Health.

[95] Dehnavieh R, Rashidian A, Maleki M, Tabibi SA, Ibrahimi Pour H, Noori Hekmat S (2011). Criteria for priority-setting in Iran basic health insurance package: exploring the perceptions of health insurance experts. HealthMED.

[96] Doshmangir L, Bazyar M, Rashidian A, Gordeev VS (2021). Iran health insurance system in transition: equity concerns and steps to achieve universal health coverage. Int J Equity Health.

[97] Kavosi Z, Rashidian A, Pourreza A, Majdzadeh R, Pourmalek F, Hosseinpour AR (2012). Inequality in household catastrophic health care expenditure in a low-income society of Iran. Health Policy Plan.

[98] Smith P, Witter S. Risk Pooling in Health Care Financing: The Implications for Health System Performance. Health, Nutrition and Population (HNP) Discussion Paper. The International Bank for Reconstruction and Development. Washington, DC: The World Bank; 2004.

[99] Khosravi B, Soltani S, Javan-Noughabi J, Faramarzi A (2017). Health care expenditure in the Islamic Republic of Iran versus other high spending countries. Med J Islam Repub Iran.

[100] Hsu J, Majdzadeh R, Harichi I, Soucat A. Health System Transformation in the Islamic Republic of Iran: An Assessment of Key Health Financing and Governance Issues. World Health Organization; 2020.

[101] Ghoddoosi-Nezhad D, Janati A, Arab-Zozani M, Doshmagir L, Sadeghi Bazargani H, Imani A (2017). Is strategic purchasing the right strategy to improve a health system’s performance? A systematic review. Bali Med J.

[102] Kutzin J, Cashin C, Jakab M. Implementing Health Financing Reform: Lessons from Countries in Transition. WHO Regional Office for Europe; 2010.

[103] World Health Organization (WHO). Health Financing Regional Profile 2018: Transitioning to Integrated Financing and Service Delivery of Priority Public Health Services. WHO; 2018.

[104] Sparkes SP, Eozenou PH, Evans D, Kurowski C, Kutzin J, Tandon A (2021). Will the quest for UHC be derailed?. Health Syst Reform.

[105] World Health Organization (WHO). The World Health Report: Health Systems Financing: The Path to Universal Coverage: Executive Summary. WHO; 2010. 10.2471/BLT.10.078741 PMC2878164 20539847

[106] Mahbobi M, Ojaghi S, Ghiasi M, Afkar A (2010). Supplemental insurances and Induced demand in chemical veterans. Iran J War Public Health.

[107] Labelle R, Stoddart G, Rice T (1994). A re-examination of the meaning and importance of supplier-induced demand. J Health Econ.

[108] Pezeshki MZ, Janati A, Arab-Zozani M (2020). Medical overuse in the Iranian healthcare system: a systematic scoping review and practical recommendations for decreasing medical overuse during unexpected COVID-19 pandemic opportunity. Risk Manag Healthc Policy.

[109] Ellenbogen MI, Wiegand AA, Austin JM, Schoenborn NL, Kodavarti N, Segal JB (2023). Reducing overuse by healthcare systems: a positive deviance analysis. J Gen Intern Med.

[110] Mosadeghrad AM, Isfahani P (2023). Strategies for reducing induced demand in the health system: a scoping review. J Health Adm.

[111] Rostami V, Shojaei P, Bahmaei J (2020). Interpretive Structural Modeling of the Factors Affecting Induced Demand for Health Services. Health Manag Inf Sci.

[112] Aghaei Hashjin A, Rajaie S. Induced demand in health: a systematic review. Strategic Studies of Public Policy 2021;11(40):440-53. [Persian].

[113] Derakhshani N, Maleki M, Pourasghari H, Azami-Aghdash S (2021). The influential factors for achieving universal health coverage in Iran: a multimethod study. BMC Health Serv Res.

[114] Gibbs T (2011). Sexy words but impotent curricula: can social accountability be the change agent of the future?. Med Teach.

[115] Heidarzadeh A, Hedayati B, Alvandi M, Rezaei M, Farrokhi B, Dadgaran I (2023). Referral system challenges of the family physician program in Iran: a systematic review. Med J Islam Repub Iran.

[116] Boelen C, Dharamsi S, Gibbs T (2012). The social accountability of medical schools and its indicators. Educ Health (Abingdon).

