# Supplementary material for: Challenges and Weaknesses of Leadership and Governance-related Health Policies in Iran: A Systematic Review
Source: Arch Iran Med. 2024 Sep 1;27(9):508–21. doi: 10.34172/aim.28907 (PMC11496596; doi:10.34172/aim.28907)
Supplement: Supplementary file 1 — contains Table S1. [file aim-27-508-s001.pdf]

Arch Iran Med. September 2024;27(9):508-S1

10.34172/aim.28907

Supplementary file 1

Table S1. Main characteristics of included studies

Citation details	Purpose of the study	Participants	Context (city)	Setting	Qualitative methodology	Study methods	Policy details
Afzali et al, 2011[33]	(i) Identifying the factors affecting hospital efficiency and (ii) identifying options for actions that would make an inefficient hospital more efficient.	Health professionals and managers	—	SSO headquarters building	NA	Content analysis- purposive sampling- in-depth interviews and FGD	Hospital efficiency
Ahmady et al, 2020[37]	Evaluating the challenges and strategies for scientific authority in medical science	Higher education experts and MOHME (Ministry of Health and Medical Education) policymakers	Tehran	Ministry of Health and Medical Education	NA	Inductive content analysis approach- Purposive sampling- FGD-	Authority in the medical science
Yazdi-Feyzabadi et al, 2015[38]	Evaluating the challenges and obstacles of high-quality health management information systems (HMIS) at the PHC system in district levels	Frontline workers (Behvarz), health technicians, district health professional, statisticians, and family physicians	Kerman province	Deputy of Health	NA	Framework analysis- purposeful sampling- semi-structured FGDs	Health information system (HIS)
Bazyar et al, 2020[39]	Assessing operational or structural challenges in what aspects of health insurance exist among health insurance schemes in	Key informants from different associations and organizations and documentary review	Tehran	Interviewees' workplace as they were comfortable there	NA	Content analysis using the 'framework method'- purposeful and snowball sampling-documentary review and semi-structured face-to-face interviews	Health insurance

	Iran which may impede or affect the adoption of the policy or its successful implementation						
Bazyar et al, 2018[40]	Revealing which actors may lose or gain benefits as a result of merging health insurance schemes in Iran	Various stakeholders including MoHME, MoCLSW, the Budget and Planning Organization, the four main public health insurance organizations, Majlis, other health insurance organizations like Petroleum Industry Health Organization, banks, Tehran Municipality, the HCHI, Iranian Medical Council, health care providers and facilities	-	NA	NA	Thematic analysis- purposive and snowball sampling- semi-structured face-to-face interviews	Merging health insurance schemes
Damari et al, 2013[41]	Assessing the performance of the boards and to adjust them to the mandates raised by new visions of the country	Members of boards of trustees and officers in charge of board's affairs at medical universities	National	Medical university or higher education/research institute under the Ministry of Health and Medical Education	Descriptive	-	Boards of Trustees of Medical Universities
Davari et al, 2012[42]	Reviewing the history of health insurance in Iran and then evaluate its performance based on the results of the interviews and empirical evidence	Healthcare delivery system, health insurance organizations and the pharmaceutical division	-	NA	NA	Thematic analysis- purposive sampling- semi-structured interviews	Health Insurance System
Doshmangir et al, 2017[43]	Exploring the infrastructures required for the implementation of	Policy makers and policy analyzers in various national and regional levels of health system,	-	MoHME,- Health insurance organizations	NA	Thematic analysis approach (inductive-deductive)- purposive and snowball (chain-referral) sampling-	Family Physician Program

	FP, without which viable implementation of the program was put in jeopardy	from organizations including MoHME, Health insurance organizations, particularly those with experiences in Family Medicine (FM) program development and implementation, health professionals and other stakeholders who influenced various processes (agenda setting, formulation, implementation and evaluation) of FM program				data collection by indexed documents, and in-depth and semi structured interviews	
Doshmangir et al, 2015[44]	Exploring the effects of targeted subsidies reform implementation on health-related behavior of population in Iran.	Policy makers and policy analysts among the highest levels of management agencies, researchers and academics. Among the households admitted to one of the major public hospitals in Tehran, 18 families (parents)	Tehran	Management agencies- faculties- hospital	NA	Content analysis (inductive-deductive)- purposive sampling- Semi structured in depth interviews	Targeted Subsidies Policy
Doshmangir et al, 2016[45]	Assessing the proposed hospital autonomy policy	Former Ministers of Health, Advisor to minister, Senior officers in medical tariff unit, Senior policy officials in health policy making council, Senior officials in Budget office, The Members of the 2nd, 3rd, 4th Parliament, Former senior national officials Former Senior policy officials in health Commission of Parliament, Member of the Managing Board in Medical Services Insurance Organization,	-	NA	NA	Content analysis (deductive-inductive)- purposeful and snowball sampling techniques- semi-structured interviews	Hospital autonomy policy

		Academic, Senior Policy Maker of the Iranian Health System, Hospital managers, Head of financial affairs of hospitals, Head of financial affairs of University, University teachers, Former Senior Policy officers, Former Head of organization, Senior Policy officials, Health policy researchers and public policy researchers					
Doshmangir et al, 2020[46]	Evaluating the experience of setting health care services tariffs in the Iranian health care system over the last five decades	Senior MoHME officials, senior management and planning organization deputies and officials, SSO officials, IMC senior policy makers, academic researchers, treatment deputy members	NA	MoHME officials	NA	Thematic framework approach- reviewing the official documents and purposive and snowball sampling- semi structured interview-	Health care services tariffs
Doshmangir et al, 2015[47]	Providing an in-depth understanding of the implementation of a model of hospital decentralization (hospital board of trustees' policy) in the Iranian health system	Health system experts including policy makers, healthcare managers, members of selected hospitals' boards of trustees, and health caregivers of the health system from MoHME, Parliament, Insurance organizatons, hospitals, Universites of Medical Sciences, Vice-Presidency for Strategic Planning and Supervision (formerly budgetng and planning organizaton) Iranian Medical Council and others, Documents from: related organizations, including: MoHME, the Parliament of the Islamic	NA	NA	NA	Thematic analysis – purposive and snowball sampling - semi structured, face-to-face interviews and two focus group discussions (involving 8 and 10 participants, respectively)	Hospital decentralization (hospital board of trustees' policy)

		Republic of Iran (Majlis), the Vice-Presidency for Strategic Planning and Supervision, the Iranian Medical Council, various insurance organizations and the internet					
Doshmangir et al, 2018[48]	Investigating aspects of the payment system in the urban FPP of the health system in the Islamic Republic of Iran	Health system experts from MoHME, medical universities, insurance companies, and FPs	NA	-	NA	Qualitative content analysis- purposive sampling- semi-structured interviews	Family physician program
Esmaeili et al, 2015[18]	Exploring the perspectives of policy-makers and decision-makers in major organizations of Iran health system about the role of family physician in the strengthening of Iran's health system for facing the current challenges	Ministry of health and medical education, Iranian Health Insurance Organization, Medical Universities and Affiliated Research Centers, Iran Medical Council, Executive Directors in Pilot Provinces	NA	NA	NA	Framework analysis- Semi structured face to face interview- purposive sampling	Family physician program
Fardid et al, 2019[49]	Assessing the challenges related to the national UFPP of Iran 4 years after its implementation in 2017	National and regional policy-makers, managers, physicians, health professionals, patients, and members of the public who actively or passively affected the process of decision-making, management, and implementation of UFPP (Urban FP Program)	National and regional level (FARS province)	NA	NA	Semi- structured and in depth interviews and focus groups - framework analysis AND content analysis of national documents - purposeful and snowball sampling-	Urban family physician program
Gorji et al, 2018[50]	Examining the problems encountered in the strategic purchasing process in Iran. The purpose of this study was to identify the problems and	General directors of the IHIO agencies, Deputy and chief executive officer of general department of health insurance, Medical records accounting, Supervision and evaluation, Financial	Tehran	-	-	Semi structured interviews, face to face interviews by telephone - framework and content analysis - Purposeful sampling	Strategic purchasing of healthcare services

	barriers which prevented the implementation of strategic purchases in IHIO(Iran Health Insurance Organization)	management, Managers of quality services, General director, Board of directors, Department of health economics, school of management and medical information					
Hassani et al, 2013[51]	Exploring the real role and place of human resource (HR) in health system reform- and determining within the whole system through the comprehensive Human Resource Management (HRM) model	Health Resource managers of universities of MoHME	NA	NA	NA	Delphi survey and a questionnaire-quantitative content analysis and expert panel and deep focus study of recorded documents-	Human Resource Management
Heydari et al, 2018[52]	Identifying and analyzing the actors of the health insurance system of Iran.	Health insurance experts	NA	All organizations in the health insurance system	NA	Stakeholder analysis model- semi-structured and structured interviews- checklist and matrixes that determined the characteristics of the stakeholders for data collection- content analysis technique - purposeful sampling	Health Insurance System (HInS)
Heydari et al, 2017[53]	Evaluating the FP model and attitudes of FPs involved toward the model	Family physicians (FPs)	Ghaleno (a county in the east of Shiraz)	NA	NA	Descriptive analysis of foregoing model and considering its goal, plan, and dimensions, and a qualitative study using in-depth and structured interview with four managers of this model in Ghaleo region as well as the family physicians (FPs) of the model- conventional content analysis-	Urban Community Health Centers in Relation to Family Physician and Primary Health Care

Ibrahimipour et al, 2011[54]	Understanding of the Iranian health financing system and providing information for policy makers about achieving universal coverage	Main actors in the Iranian health insurance system including the Ministry of Welfare and Social Security, Social Security Organization, Medical Service Insurance Organization, Army Medical Insurance Organization, Imam Khomeini Relief Foundation, Management and Planning Organization (MPO) and academics with outstanding work in the areas of health insurance and reform	NA	-	Qualitative descriptive cross-sectional study	Framework analysis- semi-structured interviews and complementary interviews (by phone)- purposeful sampling	Health insurance
Jafari et al, 2018[55]	Evaluating the barriers in policy implementation, providing solutions, and performing studies for developing more efficient regulation	Medical universities and hospitals	National	The universities and their related hospitals	Content analysis	Content analysis- survey forms - There is no mention of sampling-	Hospital board of trustees' policy
Jafari et al, 2011[56]	Exploring whether the conceptual framework proposed by the World Bank covers all aspects of hospital autonomy in Iran	Senior managers of healthcare system (i.e. hospital director, executive director, nursing manager, financial manager, human resources, and logistics manager and the hospital's deputy of education)	Tehran	General hospitals affiliated to the aforementioned universities	NA	Framework analysis technique- semi-structured interview- two hospitals randomly from each university were selected	Hospital autonomy
Kiani et al, 2021[57]	Investigating the international as well as upstream national policies and laws that support refugee's health and identified healthcare services provided	Experts from the Ministry of Health and Medical Education (MoHME), Iran's Health Insurance Organization (IHIO) and the National Welfare Organization (NWO) and	NA	-	Document review and qualitative descriptive	Semi structured interviews- purposeful sampling of key informants- thematic analysis approach-	Refugees and Sustainable Health Development

	for refugees in Iran	health care providers from both PHC and rehabilitation service					
Markazi-Moghaddam et al, 2014[58]	Exploring the obstacles of establishing autonomous hospitals in the Iranian public health sector and to figure out how the obstacles hindered the reform	Members of medical universities and members of university hospitals that had been granted autonomy in Iran	National	NA	NA	Unstructured questionnaires and semi-structured telephone interviews-thematic analysis and framework approach - purposeful sampling	Autonomous Hospitals
Mehrolhassani et al, 2013[59]	Evaluating factors affecting the implementation of accrual accounting systems in the Iranian health sector	Central Organizations of Iran Universities of Medical Sciences, Ministry of Health and Medical Education	National and provincial levels (Kerman)	KUMS	NA	Thematic framework analysis and a questionnaire with a ten-point Likert scale-descriptive indicators, principal component and factorial analyses-purposive sampling-face-to-face in-depth interviews	Accounting System Reform in Health Sector
Mohsenpour et al, 2017[60]	Exploring and analyzing the process of development and enactment of law in Iran's parliament, and factors that might affect the enactment of laws that are related to the allocation and distribution of health sector resources in Iran.	Experts of MOHME, Health commission of the parliament, Management committee of medical universities, Deputy of strategic planning of presidency, General Inspection Organization of Iran, Research center of the parliament	NA	NA	NA	Review of literature and national documents, and experts' interviews- face-to-face semi-structured interviews	Resource allocation in healthcare
Naghdi et al, 2017[61]	Investigating barriers to develop financial protection against catastrophic and impoverishing	Experts in social welfare, health care financing and health insurance	NA	NA	NA	Framework analysis-Purposeful sampling- in-depth interviews	Financial Protection

	health expenditure as a requirement to achieve universal health coverage in Iran.						
Naseriasl et al, 2018[62]	Presenting practices to address concerns about referral system in rural areas of Iran	Policy-makers of national level and experts of the health system. Family physicians (FPs), specialized physicians (SPs), and insurance organization experts were also selected based on predetermined criteria	NA	NA	NA	Content analysis- purposive approach and snowball sampling method- 28 semi-structured interviews and six sessions of focus group discussion (FGD)	Referral system
Nekoei-Mogadam et al, 2018[63]	Examining Iran's accreditation program through a case study of hospitals affiliated with the Kerman University of Medical Sciences	Experts from the university's Office of Improvement, hospital matrons, hospital managers, and experts from accreditation departments	Kerman	Hospitals affiliated with the Kerman University of Medical Sciences	NA	Purposive sampling- semi-structured interviews- framework analysis method	Hospital accreditation
Nekoei-Mogadam et al, 2013[64]	Identifying the motivations behind informal payments in Iran, how informal payments are perceived and why they are made in the health system of Iran from the perspectives of patients, health care providers and officials.	Consumers, providers and officials from public and private sectors, teaching and non-teaching facilities in Kerman province	Kerman province	NA	NA	Content analysis- face-to-face semi-structured interviews-purposeful sampling	Informal Payments in Healthcare
Parsa et al, 2015[65]	Investigating different aspects of informal payments that are also known as under-the-table payments in Iran	Surgeons and non-surgeon specialists. The surgeons consisted of otolaryngologist, urologist, general surgeon, and orthopedists and non-surgeons included	NA	NA	NA	Content analysis- purposive sampling-in depth semi structured interviews-	Informal payments

		neurologist, dermatologist, pathologist, radiologist, cardiologist and radiotherapist.					
Pourabbasi et al, 2019[66]	Evaluating the methods applied for the compilation of evolution and innovation program of medical sciences training as well as the most important directions for evolution and innovation	National macro documents included Iran's Vision Policy of 2025, documents of Supreme Council for Cultural Revolution, holistic scientific map of the country and comprehensive scientific map of health and major policies of the health system reform plan.	NA	NA	Document review	Content analysis-	National Medical Education Evolution and Innovation Plan
Poursheikhali, 2021[67]	Evaluating the current status of cooperation in Iran's PHC system from the perspective of Amour's 5 construct Model and to identify the challenges in this field.	Urban and rural family physicians, general medical physicians, and the community health workers (Behvarz)-	Kerman, East Azarbaijan, Khuzestan, Fars, and Mazandaran	NA	NA	Framework analysis using the data from 3 phases of the survey (using questionnaires of the PCET model in 5 provinces (Kerman, East Azarbaijan, Khuzestan, Fars, and Mazandaran) of Iran), expert interviews (30 experts were interviewed about the challenges of cooperation and coordination of health care providers in the primary health care system- purposeful sampling- content analysis) , and systematic review using the Amour model-	Primary Health Care System
Ravaghi et al, 2014 [68]	Assessing facilitators and barriers in CG implementation from the viewpoint of curative deputies in Iranian Medical Universities.	Deputies for curative affairs of all types of Iranian medical universities	NA	NA	NA	Qualitative thematic framework analysis - face to face interviews and relevant document reviews- purposeful sampling-	Clinical Governance
Rooddehghan et al, 2014[69]	Evaluating the perspective of	Nurses	NA	-	NA	Thematic analysis approach- purposive	Equity-Oriented Health Care

	Iranian nurses about equity in the health care system					sampling- In-depth, semi-structured interviews	System
Sabet et al, 2017[70]	Exploring pitfalls and possible challenges of urban family physician program in Iran in 2015.	Physicians enrolling in family physician program for at least 2 years:	Fasa, Shiraz	NA	NA	Conventional content analysis- purposeful sampling- semi-structured interviews	Family physician program
Sadeghi et al, 2016[71]	Explaining the strategies to develop and promote PPPs in the provision of hospital services in Iran.	Experts and professionals of the health system and individuals familiar with PPP models and roles in the development of the model in hospital services in Iran.	NA	-	NA	Content analysis- semi-structured interviews - purposive sampling	Public-private partnerships
Sajadi et al, 2016[72]	Analyzing the quantity and quality of decisions made by three governing bodies (board of trustees, board of chancellors and university councils) of a medical university of Isfahan, Iran	NA	Isfahan	Isfahan University of Medical Sciences (IUMS)	Mixed-method study	The mixed qualitative (content analysis format) and quantitative study (cross sectional format)-	Medical University's Governance
Sajadi et al, 2014[73]	Examining the perspective of board informants about the performance changes of the boards and influential factors from.	Staff and consultants who have worked with the board in Ministry of Health and Medical Education (MoHME), Ministry of Sciences and Technology and other national institutions, university's board members and the board secretaries of the universities and the vice-chancellors of the universities	NA	-	NA	**The first phase**:(qualitative research): thematic analysis - Face to face semi-structured interviews- purposive sampling **The second phase:** (a mixed qualitative part in content analysis format	Medical University's Governance
Seyedin et al, 2021[74]	Investigating factors of supplier-induced demand for	Faculty members, physicians, two public hospital managers,	NA	-	NA	Purposive sampling- semi-structured interviews- inductive content analysis	Supplier-induced demand

	health-care services in the Iranian context	patients, and researchers with academic and practical experience					for health-care services
Tabrizi et al, 2021[75]	Developing a national functional accreditation model for primary healthcare with emphasis on family practice in Iran.	Academic members in healthcare management, social medicine, and family physician fields and the experienced managers in primary healthcare field from the ministry of health and deputy of health in medical sciences universities	NA	NA	Mixed-method study	Systematic review, Delphi technique- purposeful sampling- content analysis	National Functional Accreditation Model
Yaghoubian et al, 2019[76]	Determining the requirements of strategic purchasing of health services for cancer patients and to construct a decision-making policy for insurers of Iran's health system.	Technical experts, Chief executives, Top managers	NA	NA	Grounded theory	Framework analysis - Semi structured and deep interviews- purposive and Snowball sampling	Strategic Purchasing of Health Services
Zalani et al, 2018[77]	Indicating how recent Iranian HRH strategies were developed for the Third Global Forum on Human Resources for Health in Recife,	Human resource management experts, Technical experts from MoH, human resource managers from different universities	NA	NA	NA	Content analysis- FGDs and document review- purposeful sampling-	Human resources management
Abedi et al, 2017[78]	Analyzing the patient referral system at all levels of the health system using Strengths, Weaknesses, Opportunities and Threats (SWOT) approach	Faculty members, family physicians, senior managers and health professionals	Mazandaran	NA	NA	Structure analysis and theme analysis purposive sampling- In-depth and semi-structured interview	Family Physician Plan
Abolhallaje et al, 2016[79]	Assessing the new financial system of medical universities	Financial managers and experts in Iran's health system	NA	Iran's health system	NA	Strategic planning model was investigated using the SWOT matrix	Financial System in Medical Universities

	in Iran using SWOT analysis					[strengths, weakness (internal factors), opportunities, and threats (external factors)]. Analytical hierarchy process was applied to weight and prioritize the items.	
Anjomshoa et al2021[27]	Evaluating health financing system progress towards achieving UHC in Iran	Health financing experts, policy-makers and academics	Tehran	Department of Health Management and Economics, School of Public Health, Tehran	Explanatory mixed-method study	-	UHC in Iran
Doshmangir et al, 2019[80]	Policy analysis of the Iranian Health Transformation Plan in primary healthcare	Representatives from universities, researchers, academics, and policy makers at the micro, meso and macro levels at the MoHME, health insurance organizations, Iran Medical Council, Parliament and Academy of Medical Sciences	Iran	Iran National Institute of Health Research	The qualitative study design (using multiple methods)	Document analysis, round-table discussion, and semi-structured interviews.	Iranian Health Transformation Plan in primary healthcare
Falahat et al, 2013[81]	Evaluating governance in community based health programmes	Comprising CBHP managers, researchers, WHO representatives in Iran and a number of programme managers from the Ministry of Health and Medical Education (MOHME)	Various locations in Iran	NA	NA	Individual interviews, Focus group discussions	Advocate more friendly policies in community, academy and funding organizations
Farzadfar et al, 2017[82]	Assessing problems in implementation of urban family physician program in Iran	Physicians, midwives in public and private system, health managers	Alborz, West Azerbaijan, and Kurdistan	Health system	NA	Individual interviews, Focus group discussions	Urban family physician program
Khankeh et al, 2020[83]	Evaluating healthcare System Reform in Iran	High-ranking policymakers and healthcare providers at the national and provincial levels	Shiraz	Iran's health system	NA	Macro-qualitative study- by interviewing experienced individuals	Healthcare System Reform

Marnani, 2010[84]	Assessing health Insurance Organization	Senior managers of one of the largest health insurance organizations	Tehran	Insurance organizations	An applied and qualitative research	Structured interviews and deep interviews	Health insurance coverage
Mohammadpour et al, 2020[85]	Evaluating oral health policymaking challenges	Experts who had experience in oral health	Shiraz	Shiraz University of Medical Sciences	Qualitative study	Snowball sampling- semi-structured interviews	Oral health policymaking
Moshiri et al, 2016[86]	Analyzing policy of the development and implementation of the PHC program, as well as situation of the referral system in Iran	Former and present senior MOH officials, faculty members, provincial health managers, community health workers and two of the former health ministers	Tehran and six other cities	NA	Qualitative study	A policy analysis using the policy triangle framework developed by Walt and Gilson	Primary health care in Iran
Nejatzadegan et al, 2016[87]	Challenges in the rural family doctor system in Iran	Family physicians and 13 executive directors of health	Eastern Iran in a region with an area of 118.854 km2 and a population of 5994402	Health system	Qualitative study	Snowball sampling- conceptual framework method	Rural family physician program in the east of Iran
Safizadehe et al, 2016[88]	Evaluating patient referral system in urban family physician program	Administrative officers, family physicians, executive managers, and individuals working in insurance sector and people receiving insurance services	Mazandaran	Iran's health system	Qualitative study	Semi-structured and in-depth interview. content and structural analysis	Urban Family Physician Program
Sarvestani et al, 2017[70]	Exploring pitfalls and possible challenges of urban family physician program in Iran	physicians enrolled in the family physician program	Fasa, Shiraz	Health centers	Descriptive exploratory design	Content analysis approach-semi-structured interviews	Urban Family Physician Program
Takian et al, 2011[89]	Assessing the facilitators and barriers to FM implementation	Different stakeholders including policy makers at the national level, managers and officials at provincial level, and practitioners, managers and public representatives at local level	Iran	Iran's health system	A qualitative design	Semi-structured interviews-purposeful sampling-narrative analysis of selected documents.	FM implementation
